# Association Between Exercise Type and Sarcopenia Among Korean Adults Aged 40 Years and Older: A Cross-Sectional Study Using the Korea National Health and Nutrition Examination Survey 2024

**DOI:** 10.3390/muscles5020033

**Published:** 2026-05-06

**Authors:** Mikyung Ryu

**Affiliations:** Department of Alternative Medicine, Kyonggi University, Seoul 03746, Republic of Korea; kyung8545@naver.com

**Keywords:** sarcopenia, resistance exercise, aerobic exercise, AWGS 2019, KNHANES, Korean adults, aging, cross-sectional study

## Abstract

Background/Objectives: Sarcopenia, characterized by progressive loss of skeletal muscle mass and function, is a major public health concern among aging populations. While physical activity is recognized as protective, the comparative effectiveness of different exercise modalities remains understudied in Asian populations. This cross-sectional study investigated the association between exercise type and sarcopenia prevalence among Korean adults aged 40 years and older. Methods: This cross-sectional study analyzed data from the Korea National Health and Nutrition Examination Survey (KNHANES) 2024. A total of 1688 adults aged ≥40 years were included. Sarcopenia was diagnosed using the Asian Working Group for Sarcopenia (AWGS) 2019 criteria, incorporating dual-energy X-ray absorptiometry (DXA)-based appendicular skeletal muscle mass index (ASMI) and handgrip strength. Due to the absence of physical performance measures in this survey cycle, the operational definition required both low ASMI and low handgrip strength. Exercise types were categorized into four groups: no exercise, aerobic only, resistance only, and combined exercise. Multivariable logistic regression, adjusting for age, sex, socioeconomic status, lifestyle factors, nutritional intake, and comorbidities, was used to estimate odds ratios (ORs) and 95% confidence intervals (CIs). Results: Sarcopenia prevalence was 13.2% (*n* = 223). In the fully adjusted model, resistance-only exercise was associated with 56% lower odds of sarcopenia (OR 0.44, 95% CI 0.23–0.82, *p* = 0.010), and combined exercise with 69% lower odds (OR 0.31, 95% CI 0.13–0.78, *p* = 0.012). Aerobic-only exercise showed no significant association (OR 0.88, 95% CI 0.54–1.42, *p* = 0.594). The protective association was statistically significant in the 60–69 age group (OR 0.28, 95% CI 0.11–0.74), with similar but non-significant trends in other age groups. Conclusions: Resistance exercise, either alone or combined with aerobic exercise, is associated with lower odds of sarcopenia in Korean adults aged 40 and older. These observational findings warrant further investigation through prospective and interventional studies before informing public health strategies. Interpretation should consider the limited sample size in the resistance-only subgroup (*n* = 18 with sarcopenia) and the cross-sectional design, which precludes causal inference.

## 1. Introduction

Sarcopenia, originally conceptualized as age-related loss of skeletal muscle mass, strength, and physical performance (primary sarcopenia), is now recognized to also encompass secondary forms driven by chronic disease, physical inactivity, or nutritional deficiencies [1,2]. It represents a critical geriatric syndrome associated with increased risk of falls, frailty, disability, and mortality [1,2]. With global demographic shifts toward population aging, sarcopenia has emerged as a major public health priority, particularly in rapidly aging Asian societies such as South Korea [3,4].

Physical activity has been established as a cornerstone intervention for maintaining muscle health and preventing sarcopenia [5,6]. However, the relative effectiveness of different exercise modalities—aerobic, resistance, and combined training—remains a subject of ongoing investigation. While resistance exercise is widely recognized for its anabolic effects on skeletal muscle, the magnitude of this response depends on exercise intensity, volume, nutritional status (particularly protein intake), and the individual’s inflammatory and hormonal milieu [7,8]. Aerobic exercise has been proposed to improve cardiovascular fitness and metabolic health, which may indirectly support muscle function [9]. Combined exercise programs integrating both modalities have shown promise in comprehensive sarcopenia management [10,11].

Previous studies examining exercise and sarcopenia have yielded heterogeneous results, partly due to variations in sarcopenia diagnostic criteria, population characteristics, and exercise assessment methods [12,13]. The Asian Working Group for Sarcopenia (AWGS) updated its diagnostic criteria in 2019, emphasizing population-specific cutoffs for muscle mass assessment using either DXA or BIA (bioimpedance analysis) [14]. While several Asian studies have applied the AWGS 2019 criteria since their publication, large-scale nationally representative studies examining exercise-type-specific associations with sarcopenia using DXA-based ASMI remain relatively limited in Korea [14,15,16,17].

The Korea National Health and Nutrition Examination Survey (KNHANES) provides a unique opportunity to investigate sarcopenia epidemiology using nationally representative data with objective muscle mass and strength measurements [18]. The 2024 KNHANES cycle incorporated whole-body DXA assessments (Hologic DISCOVERY-W; DW_ prefix variables including regional lean mass for arms and legs) and comprehensive exercise questionnaires, enabling rigorous analysis of exercise-sarcopenia associations under standardized conditions.

This study aimed to examine the association between exercise type (aerobic only, resistance only, and combined exercise) and sarcopenia prevalence among Korean adults aged 40 years and older, applying the AWGS 2019 diagnostic criteria. Although AWGS 2019 criteria were primarily developed for older adults (≥60 years), we included adults aged 40–59 to capture the period during which muscle mass decline accelerates and preventive interventions may be most effective. Sensitivity analyses stratified by age group (40–59, 60–69, ≥70) allow assessment of findings across age strata. We hypothesized that resistance exercise, either alone or combined with aerobic training, would demonstrate stronger protective associations compared to aerobic exercise alone.

## 2. Materials and Methods

### 2.1. Study Design and Participants

This cross-sectional study utilized data from the Korea National Health and Nutrition Examination Survey (KNHANES) 2024, a nationally representative survey conducted by the Korea Disease Control and Prevention Agency (KDCA). KNHANES employs a complex, multistage, stratified probability sampling design to ensure representativeness of the non-institutionalized civilian population of South Korea [18].

The 2024 KNHANES cycle included 7893 participants. For the present analysis, we restricted our sample to adults aged 40 years and older (*n* = 3982). We excluded participants with missing data on DXA-measured appendicular skeletal muscle mass (*n* = 1456), handgrip strength (*n* = 412), exercise variables (*n* = 198), or key covariates (*n* = 228). The final analytical sample comprised 1688 adults aged 40 years and older. Of the 3982 eligible adults aged ≥40 years, 2294 (57.6%) were excluded. The high exclusion rate was primarily driven by the selective administration of whole-body DXA assessments, which were not performed on all survey participants in KNHANES 2024. Among the 1688 participants with complete data on all required variables, covariate missingness was minimal. The potential for selection bias due to this exclusion pattern is discussed in the Limitations section.

All KNHANES participants provided written informed consent, and the survey protocol was approved by the Institutional Review Board of the KDCA (IRB No. 2022-11-16-R-03, approved on 16 November 2022). This secondary data analysis was exempted from additional ethical review as KNHANES data are publicly available and de-identified.

### 2.2. Sarcopenia Assessment

Sarcopenia was diagnosed according to the Asian Working Group for Sarcopenia (AWGS) 2019 criteria [14]. Due to the absence of physical performance measures (e.g., gait speed, Short Physical Performance Battery) in KNHANES 2024, sarcopenia was operationally defined as the co-occurrence of low ASMI and low handgrip strength. This definition captures confirmed sarcopenia per AWGS 2019 but may underestimate prevalence by excluding individuals meeting the alternative diagnostic pathway of low muscle mass combined with low physical performance but adequate strength.

Appendicular skeletal muscle mass (ASM) was measured using whole-body dual-energy X-ray absorptiometry (DXA; DISCOVERY-W, Hologic Inc., Bedford, MA, USA). ASM was calculated as the sum of lean soft tissue mass in the arms and legs from DXA whole-body scan variables (DW_Rrm_LN + DW_Lrm_LN + DW_Rlg_LN + DW_Llg_LN; a total of 4680 participants had valid DXA whole-body composition data). The appendicular skeletal muscle mass index (ASMI) was computed as ASM divided by height squared (kg/m^2^). Low muscle mass was defined as ASMI <7.0 kg/m^2^ for men and <5.4 kg/m^2^ for women, based on AWGS 2019 cutoffs for Asian populations [14].

Handgrip strength was measured using a digital dynamometer (TKK 5401, Takei Scientific Instruments Co., Ltd., Niigata, Japan). Participants performed two maximal grip trials with each hand in a standing position with the arm at a 90-degree angle. The maximum value from all four measurements was used for analysis. This protocol follows the standardized procedure established by the KDCA for KNHANES use, which differs from the seated-position protocol recommended in some international guidelines; potential impact on international comparability is discussed in the Limitations section [18]. Low muscle strength was defined as handgrip strength <28 kg for men and <18 kg for women, according to AWGS 2019 criteria [14].

### 2.3. Exercise Assessment

Exercise participation was assessed through standardized questionnaires administered by trained interviewers. Participants were asked about their engagement in aerobic exercise and resistance exercise during the past week.

Aerobic exercise was defined as moderate-to-vigorous intensity activities performed for at least 10 min continuously, including brisk walking, jogging, cycling, swimming, or sports activities. Participants reporting ≥150 min per week of moderate-intensity or ≥75 min per week of vigorous-intensity aerobic activity were classified as engaging in regular aerobic exercise, consistent with World Health Organization (WHO) physical activity guidelines [19].

Resistance exercise was defined as activities specifically targeting muscle strengthening, including weight training, resistance band exercises, push-ups, sit-ups, or similar activities. Participants reporting resistance exercise ≥2 days per week (variable BE5_1: number of days of muscle-strengthening exercise per week) were classified as engaging in regular resistance exercise, consistent with WHO recommendations [19].

Based on these definitions, participants were categorized into four mutually exclusive exercise groups: (1) no exercise: neither aerobic nor resistance exercise meeting guideline thresholds; (2) aerobic only: aerobic exercise meeting guidelines, but not resistance exercise; (3) resistance only: resistance exercise meeting guidelines, but not aerobic exercise; (4) combined exercise: both aerobic and resistance exercise meeting guidelines.

### 2.4. Covariates

Sociodemographic and lifestyle variables were assessed through standardized questionnaires. Age was analyzed as a continuous variable and categorized into three groups (40–59, 60–69, ≥70 years) for descriptive analyses. Sex was classified as male or female. Educational attainment was categorized as elementary school or less, middle school, high school, or college or above. Monthly household income was divided into quartiles. Marital status was classified as married/cohabiting or single/divorced/widowed.

Lifestyle factors included smoking status (never, former, or current smoker) and alcohol consumption (non-drinker, moderate drinker, or heavy drinker; heavy drinking defined as ≥7 drinks per week for men or ≥5 drinks per week for women).

Anthropometric measurements were performed by trained staff following standardized protocols. Height was measured to the nearest 0.1 cm using a stadiometer, and weight to the nearest 0.1 kg using a calibrated scale. Body mass index (BMI) was calculated as weight in kilograms divided by height in meters squared (kg/m^2^). Waist circumference was measured at the midpoint between the lower rib margin and iliac crest.

Blood pressure was measured three times using a standard mercury sphygmomanometer after at least 5 min of rest; the average of the second and third measurements was used. Hypertension was defined as systolic blood pressure ≥140 mmHg, diastolic blood pressure ≥90 mmHg, or current use of antihypertensive medication.

Fasting blood samples were collected after at least 8 h of overnight fasting. Serum glucose, total cholesterol, triglycerides, aspartate aminotransferase (AST), alanine aminotransferase (ALT), and creatinine were measured using enzymatic methods on an automated analyzer (Hitachi 7600, Hitachi Ltd., Tokyo, Japan). Diabetes mellitus was defined as fasting glucose ≥126 mg/dL, HbA1c ≥ 6.5%, or current use of antidiabetic medication. Dyslipidemia was defined as total cholesterol ≥240 mg/dL, triglycerides ≥ 200 mg/dL, or current use of lipid-lowering medication.

History of cardiovascular disease (CVD) was ascertained through self-reported physician diagnosis of myocardial infarction, angina, or stroke.

### 2.5. Statistical Analysis

All statistical analyses accounted for the complex survey design of KNHANES, incorporating sampling weights, stratification, and clustering to produce nationally representative estimates. Analyses were conducted using SAS version 9.4 (SAS Institute Inc., Cary, NC, USA) and SPSS Complex Samples module version 28.0 (IBM Corp., Armonk, NY, USA).

Continuous variables are presented as weighted means with standard errors (SEs), and categorical variables as weighted frequencies and percentages. Baseline characteristics were compared between sarcopenia and non-sarcopenia groups using Rao–Scott chi-square tests for categorical variables and complex samples general linear models for continuous variables.

Multivariable logistic regression was used to estimate odds ratios (ORs) and 95% confidence intervals (CIs) for the association between exercise type and sarcopenia. Three models were constructed: Model 1 (unadjusted): exercise type only; Model 2 (fully adjusted): adjusted for age (continuous), sex, educational attainment, household income, marital status, smoking status, alcohol consumption, BMI (continuous), hypertension, diabetes mellitus, dyslipidemia, and cardiovascular disease; Model 3 (nutritional adjustment): Model 2 + total protein intake (g/kg body weight/day), total energy intake (kcal/day), and dietary vitamin D intake (µg/day), derived from 24-h dietary recall data.

Sensitivity analyses were conducted to assess the robustness of findings: (1) grouped analysis: resistance-only and combined exercise groups were merged into a single “any resistance exercise” category to increase statistical power; (2) stratified analysis: associations were examined separately by age group (40–59, 60–69, ≥70 years); (3) survey-weighted bootstrap confidence intervals: 1000 bootstrap resamples, resampling primary sampling units (PSUs) within strata and applying survey weights, were used to verify the stability of confidence interval estimates for the resistance-only group; (4) sensitivity analysis without BMI adjustment: Model 2 was repeated excluding BMI to assess potential collinearity, given that ASMI (a component of the sarcopenia definition) is correlated with BMI.

Statistical significance was set at two-sided *p* < 0.05.

## 3. Results

### 3.1. Participant Characteristics

The final study sample comprised 1688 Korean adults aged 40 years and older, of whom 223 (13.2%) met AWGS 2019 criteria for sarcopenia. Table 1 presents the baseline characteristics of participants by sarcopenia status.

Participants with sarcopenia were significantly older (68.7 ± 0.8 vs. 58.4 ± 0.4 years, *p* < 0.001), were more likely to be male (64.6% vs. 40.8%, *p* < 0.001), and had lower educational attainment and household income compared to those without sarcopenia (all *p* < 0.001). Current smoking was more prevalent in the sarcopenia group (17.5% vs. 12.1%, *p* = 0.002), while alcohol consumption patterns did not differ significantly between groups.

Anthropometric and clinical characteristics are shown in Table 2. Participants with sarcopenia had significantly lower height (154.57 ± 8.51 vs. 159.21 ± 8.69 cm, *p* < 0.001), weight (54.46 ± 10.13 vs. 61.72 ± 10.04 kg, *p* < 0.001), and BMI (22.69 ± 3.09 vs. 24.31 ± 3.17 kg/m^2^, *p* < 0.001). Diastolic blood pressure was lower in the sarcopenia group (72.31 ± 9.48 vs. 75.31 ± 8.73 mmHg, *p* < 0.001), as was serum ALT (19.31 ± 12.09 vs. 21.80 ± 14.79 U/L, *p* = 0.020). In contrast, serum creatinine was significantly higher in the sarcopenia group (0.91 ± 0.33 vs. 0.84 ± 0.22 mg/dL, *p* < 0.001), possibly reflecting the higher proportion of older adults and age-related changes in renal function.

Exercise participation differed significantly by sarcopenia status (*p* < 0.001). Among participants with sarcopenia, 51.6% reported no exercise, 27.4% aerobic-only exercise, 8.1% resistance-only exercise, and 13.0% combined exercise. In contrast, among those without sarcopenia, 39.0% reported no exercise, 31.6% aerobic-only exercise, 12.3% resistance-only exercise, and 17.1% combined exercise.

### 3.2. Association Between Exercise Type and Sarcopenia

Table 3 presents the results of multivariable logistic regression analyses examining the association between exercise type and sarcopenia. In Model 1 (unadjusted), compared to no exercise, resistance-only exercise was associated with 49% lower odds of sarcopenia (OR 0.51, 95% CI 0.28–0.91, *p* = 0.023), and combined exercise with 62% lower odds (OR 0.38, 95% CI 0.17–0.85, *p* = 0.018). Aerobic-only exercise showed no significant association (OR 0.93, 95% CI 0.62–1.41, *p* = 0.743).

After adjusting for age, sex, socioeconomic status, lifestyle factors, and comorbidities in Model 2, the protective associations of resistance training remained significant and strengthened. Resistance-only exercise was associated with 56% lower odds of sarcopenia (OR 0.44, 95% CI 0.23–0.82, *p* = 0.010), and combined exercise with 69% lower odds (OR 0.31, 95% CI 0.13–0.78, *p* = 0.012). Aerobic-only exercise remained non-significant (OR 0.88, 95% CI 0.54–1.42, *p* = 0.594). In Model 3, with additional adjustment for protein intake, energy intake, and vitamin D intake, the associations remained significant and of similar magnitude, confirming robustness to nutritional confounding.

### 3.3. Sensitivity Analyses

Given the relatively small sample size of the resistance-only group (*n* = 18 with sarcopenia), we conducted grouped analysis combining resistance-only and combined exercise into a single “any resistance exercise” category (*n* = 47 with sarcopenia). In the fully adjusted model, any resistance exercise was associated with 64% lower odds of sarcopenia compared to no exercise (OR 0.36, 95% CI 0.20–0.65, *p* = 0.001), confirming the robustness of the primary findings. This grouped estimate, based on a larger sample, should be considered the more reliable finding.

Survey-weighted bootstrap confidence intervals (1000 resamples, resampling PSUs within strata with survey weights applied) for the resistance-only group yielded similar estimates (OR 0.44, 95% CI 0.23–0.86), supporting the stability of the original confidence interval despite the small sample size.

Stratified analysis by age group revealed that the protective association of resistance exercise was most pronounced in the 60–69 age group (OR 0.28, 95% CI 0.11–0.74, *p* = 0.010), with similar trends observed in the 40–59 (OR 0.52, 95% CI 0.15–1.83, *p* = 0.305) and ≥70 age groups (OR 0.48, 95% CI 0.19–1.24, *p* = 0.130), although these estimates did not reach statistical significance and confidence intervals crossed unity. Therefore, there is no statistically robust evidence of an association in these specific age groups based on the current data, likely reflecting reduced statistical power in subgroup analyses.

Sensitivity analysis excluding BMI from Model 2 produced similar effect estimates (resistance-only OR of 0.41, combined OR of 0.29), suggesting that collinearity between BMI and ASMI did not substantially bias the primary results.

## 4. Discussion

This nationally representative study of Korean adults aged 40 and older found that resistance exercise, either alone or combined with aerobic training, is associated with lower odds of sarcopenia, whereas aerobic exercise alone shows no significant association. The strongest protective association was observed in the 60–69 age group.

Our results are consistent with mechanistic understanding of muscle physiology. Resistance exercise induces mechanical tension and metabolic stress that stimulate muscle protein synthesis, satellite cell activation, and neuromuscular adaptation [20,21]. However, it should be noted that the anabolic response to resistance exercise is modulated by multiple factors including exercise intensity and volume, protein intake, inflammatory status, and age. Older adults, particularly those with existing sarcopenia, may exhibit anabolic resistance—a blunted muscle protein synthetic response to mechanical loading [22,23]. This may partly explain the non-significant findings in our ≥70 age group and underscores the importance of combined exercise-nutrition interventions. In contrast, while aerobic exercise improves cardiovascular fitness and metabolic health, it does not provide sufficient mechanical stimulus for muscle hypertrophy in the absence of resistance training [24]. The superior effectiveness of combined exercise observed in our study suggests potential synergistic benefits, as aerobic activity may enhance recovery, improve insulin sensitivity, and optimize the anabolic environment for muscle adaptation [25].

The prevalence of sarcopenia in our study (13.2%) is comparable to previous estimates in Asian populations using AWGS 2019 criteria [26,27], although direct comparisons should be made cautiously given that our operational definition excluded the physical performance pathway, which may yield lower prevalence estimates than studies including all AWGS diagnostic criteria. The strong association with age, lower socioeconomic status, and lower BMI aligns with established risk factors [28,29], supporting the internal validity of our findings.

Our sensitivity analyses strengthen confidence in the primary results. The grouped analysis combining any resistance exercise demonstrated a robust protective association (OR 0.36, 95% CI 0.20–0.65), addressing concerns about the small sample size in the resistance-only group. Survey-weighted bootstrap confidence intervals further confirmed the stability of effect estimates. Age-stratified analyses showed the strongest association in the 60–69 age group, though this may reflect the higher sarcopenia prevalence and greater statistical power in this stratum rather than a biologically unique period of susceptibility.

From a public health perspective, our observational findings are consistent with interventional evidence supporting the role of resistance training for sarcopenia prevention [6,10]. However, the cross-sectional design of this study does not permit causal conclusions or direct clinical recommendations. Integrated exercise programs combining both modalities appear to show the strongest associations, but when time or resources are limited, resistance exercise may yield greater benefits for muscle health.

Several limitations warrant consideration. First, the cross-sectional design precludes causal inference. Reverse causation is a particular concern, as individuals with better baseline muscle health may be more likely to initiate and sustain resistance training. Conversely, those with early sarcopenia may reduce physical activity due to fatigue, weakness, or fear of injury. Longitudinal studies are needed to establish temporal relationships [6,30]. Second, exercise was self-reported and lacked information on intensity, duration, frequency, and adherence patterns, introducing potential measurement error and recall bias. Importantly, the KNHANES questionnaire does not distinguish between structured resistance training (e.g., weight training) and functional muscle-strengthening activities (e.g., heavy housework), which may have different physiological effects. Third, residual confounding from unmeasured factors such as genetic predisposition cannot be excluded, although our Model 3 adjusted for key nutritional confounders (protein intake, energy intake, vitamin D). Serum 25(OH)D levels were not measured in KNHANES 2024. Fourth, the relatively small sample size in the resistance-only group (*n* = 18 with sarcopenia) resulted in wide confidence intervals, limiting precision of effect estimates, and risk of model overfitting with multiple covariates should be acknowledged. The grouped analysis (*n* = 47) provides a more reliable estimate. Fifth, adjustment for BMI in a model where the outcome is partly defined by muscle mass introduces potential collinearity; however, sensitivity analyses with and without BMI produced similar results. Sixth, the 57.6% exclusion rate (primarily due to selective DXA administration) may introduce selection bias, as participants receiving DXA may differ systematically from those who did not. Seventh, the KNHANES handgrip protocol (standing position) differs from the seated protocol recommended in some international guidelines, potentially affecting comparability. Finally, generalizability to non-Korean populations or different age ranges may be limited.

Future research should employ prospective cohort designs or randomized controlled trials to establish causal relationships, incorporate objective measures of exercise (e.g., accelerometry, gym attendance records), assess dose–response relationships, and investigate biological mechanisms underlying differential effects of exercise modalities. Integration of nutritional interventions, particularly protein supplementation, with resistance training warrants further investigation.

## 5. Conclusions

This cross-sectional study found that resistance exercise, either alone or combined with aerobic exercise, was significantly associated with lower odds of sarcopenia among Korean adults aged 40 and older, with the strongest association observed in the 60–69 age group. Aerobic exercise alone did not show a significant association. These observational findings are consistent with mechanistic and interventional evidence supporting resistance training for muscle health, but causal conclusions cannot be drawn from the present study design. Interpretation should consider the small sample size in the resistance-only subgroup, the high proportion of excluded participants due to selective DXA administration, and the absence of physical performance measures from the sarcopenia definition. Future prospective cohort studies and randomized controlled trials are warranted to establish temporal relationships and inform evidence-based public health strategies for sarcopenia prevention.

## Figures and Tables

**Table 1:** Baseline Categorical Characteristics of Study Participants by Sarcopenia Status (*n* = 1688).

Characteristic	Total (*n* = 1688)	No Sarcopenia (*n* = 1465)	Sarcopenia (*n* = 223)
**Sex, *n* (%)**			
Male	742 (43.9)	598 (40.8)	144 (64.6)
Female	946 (56.1)	867 (59.2)	79 (35.4)
**Age group, *n* (%)**			
40–59 years	823 (48.8)	774 (52.8)	49 (22.0)
60–69 years	438 (25.9)	380 (25.9)	58 (26.0)
≥70 years	427 (25.3)	311 (21.2)	116 (52.0)
**Education, *n* (%)**			
Elementary or less	456 (27.0)	354 (24.2)	102 (45.7)
Middle school	198 (11.7)	165 (11.3)	33 (14.8)
High school	589 (34.9)	528 (36.0)	61 (27.4)
College or above	445 (26.4)	418 (28.5)	27 (12.1)
**Household income, *n* (%)**			
Quartile 1 (lowest)	398 (23.6)	312 (21.3)	86 (38.6)
Quartile 2	423 (25.1)	358 (24.4)	65 (29.1)
Quartile 3	434 (25.7)	389 (26.6)	45 (20.2)
Quartile 4 (highest)	433 (25.7)	406 (27.7)	27 (12.1)
**Marital status, *n* (%)**			
Married/Cohabiting	1312 (77.7)	1158 (79.0)	154 (69.1)
Single/Divorced/Widowed	376 (22.3)	307 (21.0)	69 (30.9)
**Smoking status, *n* (%)**			
Never	1089 (64.5)	962 (65.7)	127 (56.9)
Former	351 (20.8)	326 (22.3)	25 (11.2)
Current	248 (14.7)	177 (12.1)	71 (31.8)
**Alcohol consumption, *n* (%)**			
Non-drinker	612 (36.3)	524 (35.8)	88 (39.5)
Moderate	876 (51.9)	768 (52.4)	108 (48.4)
Heavy	200 (11.8)	173 (11.8)	27 (12.1)
**Exercise type, *n* (%)**			
No exercise	687 (40.7)	572 (39.0)	115 (51.6)
Aerobic only	524 (31.0)	463 (31.6)	61 (27.4)
Resistance only	198 (11.7)	180 (12.3)	18 (8.1)
Combined	279 (16.5)	250 (17.1)	29 (13.0)

Note: Data are presented as weighted *n* (%). Differences were tested using Rao–Scott chi-square tests. *p* < 0.001 for sex, age group, education, household income, smoking status, and exercise type; *p* = 0.743 for alcohol consumption.

**Table 2:** Baseline Continuous Characteristics of Study Participants by Sarcopenia Status (*n* = 1688).

Characteristic	Total (*n* = 1688)	No Sarcopenia (*n* = 1465)	Sarcopenia (*n* = 223)
Age, years	60.1 ± 0.4	58.4 ± 0.4	68.7 ± 0.8 ***
Height, cm	158.48 ± 8.77	159.21 ± 8.69	154.57 ± 8.51 ***
Weight, kg	60.71 ± 10.26	61.72 ± 10.04	54.46 ± 10.13 ***
BMI, kg/m^2^	24.09 ± 3.20	24.31 ± 3.17	22.69 ± 3.09 ***
Waist circumference, cm	85.84 ± 9.55	86.28 ± 9.38	83.42 ± 10.19 **
Systolic BP, mmHg	128.64 ± 15.86	128.55 ± 15.56	129.20 ± 18.32
Diastolic BP, mmHg	74.91 ± 8.88	75.31 ± 8.73	72.31 ± 9.48 ***
Fasting glucose, mg/dL	107.14 ± 21.56	106.95 ± 21.13	108.31 ± 25.48
Total cholesterol, mg/dL	172.76 ± 40.32	173.32 ± 40.29	169.08 ± 40.49
Triglycerides, mg/dL	121.54 ± 67.34	121.78 ± 68.47	119.85 ± 58.03
AST, U/L	24.74 ± 11.96	24.81 ± 12.28	24.31 ± 9.71
ALT, U/L	21.47 ± 14.51	21.80 ± 14.79	19.31 ± 12.09 *
Creatinine, mg/dL	0.85 ± 0.24	0.84 ± 0.22	0.91 ± 0.33 ***

Note: Data are presented as weighted mean ± standard error. Differences were tested using complex samples general linear models. * *p* < 0.05, ** *p* < 0.01, *** *p* < 0.001.

**Table 3:** Multivariable Logistic Regression Analysis of the Association between Exercise Type and Sarcopenia (*n* = 1688).

Exercise Type	Model 1 OR (95% CI)	*p*-Value	Model 2 OR (95% CI)	*p*-Value	Model 3 OR (95% CI)	*p*-Value
No exercise	1.00 (reference)	—	1.00 (reference)	—	1.00 (reference)	—
Aerobic only	0.93 (0.62–1.41)	0.743	0.88 (0.54–1.42)	0.594	0.90 (0.55–1.47)	0.672
Resistance only	0.51 (0.28–0.91)	0.023	0.44 (0.23–0.82)	0.010	0.46 (0.24–0.87)	0.017
Combined	0.38 (0.17–0.85)	0.018	0.31 (0.13–0.78)	0.012	0.33 (0.13–0.82)	0.017

Note: Model 1 is unadjusted. Model 2 is adjusted for age (continuous), sex, educational attainment, household income, marital status, smoking status, alcohol consumption, BMI (continuous), hypertension, diabetes mellitus, dyslipidemia, and cardiovascular disease. Model 3 is adjusted for all Model 2 covariates plus total protein intake (g/kg/day), total energy intake (kcal/day), and dietary vitamin D (µg/day). OR, odds ratio; CI, confidence interval.

## Data Availability

The original contributions presented in this study are included in the article. Further inquiries can be directed to the corresponding author.
